# Transcriptomic evidence of cytokine storm and sepsis in little brown bats exposed to white-nose syndrome

**DOI:** 10.1093/conphys/coaf040

**Published:** 2025-07-01

**Authors:** Robert E Kwait, Evan A Eskew, Malin L Pinsky, Sarah A Gignoux-Wolfsohn, Maarten J Vonhof, Brooke Maslo

**Affiliations:** Department of Ecology, Evolution and Natural Resources, Rutgers University, 14 College Farm Road, New Brunswick, NJ 08901, USA; Institute for Interdisciplinary Data Sciences, University of Idaho, 875 Perimeter Drive, Moscow, ID 83844, USA; Department of Ecology & Evolutionary Biology, University of California Santa Cruz, 130 McAllister Way, Santa Cruz, CA 95060, USA; Department of Biological Sciences, University of Massachusetts Lowell, 198 Riverside Street, Lowell, MA 01854, USA; Department of Biological Sciences and School of Environment, Geography, and Sustainability, Western Michigan University, 1903 W Michigan Avenue, Kalamazoo, MI 49008, USA; Department of Ecology, Evolution and Natural Resources, Rutgers University, 14 College Farm Road, New Brunswick, NJ 08901, USA

**Keywords:** Bats, gene expression, immune dysregulation, immune response, inflammation, pathophysiology, *Pseudogymnoascus destructans*

## Abstract

Much progress has been made in understanding the pathophysiology of white-nose syndrome (WNS), a devastating disease that has impacted North American hibernating bats for nearly two decades. Growth of the causative fungal pathogen, *Pseudogymnoascus destructans,* on exposed epidermal tissue of bats creates an immune reaction that disrupts natural hibernation physiology and leads to premature expenditure of energy reserves and often death. Past work has highlighted the similarities between WNS and immune reconstitution inflammatory syndrome, but other conditions that have not been considered yet may also be relevant. We performed a transcriptomic analysis of wing tissue from naïve and exposed bats to further investigate the implications of observed differential gene expression patterns. For this analysis, we collected wing biopsy samples from 41 individuals prior to WNS emergence and 58 individuals 2–5 years after WNS emergence. We generated poly-A enriched tag-Seq libraries to compare gene expression between these groups. We then linked our findings and those of past studies to other disease systems to build hypotheses regarding mechanisms of WNS pathophysiology. We found an overrepresentation of functions related to programmed cell death and cytokine activity among upregulated genes. Importantly, we also identified upregulation of three S100 damage-associated molecular patterns (DAMPs) in exposed populations. Taken together, our findings and those of past studies suggest that infected bats experience a feedback loop of cell death among immune cells, the release of DAMPs and the stimulation of cytokine release that may act to maintain pathological immune activity. This feedback loop likely relates to cytokine storms in individuals with severe infection and possibly deteriorates into sepsis over time. Given the pathophysiology of sepsis, multiple organ dysfunction potentially contributes to the physiological disruption associated with WNS.

## Introduction

Effective wildlife disease management requires an understanding of disease pathophysiology to improve the prognosis of affected individuals. However, the study of pathophysiology in wild populations can be exceedingly difficult due to the logistical challenges of capturing and housing non-domestic animals. In these cases, it may be useful to search for parallels between emerging wildlife diseases and extensively studied systems, such as diseases in domesticated animals and humans. Such connections may be especially meaningful in explaining disease trends that exist across multiple taxa. Transcriptomic analyses of several multispecies diseases have revealed such a trend (Eskew *et al*., 2021).

A common pattern of high immune reactivity in disease-susceptible species compared to more resistant species has emerged among analyses of gene expression in several taxa, with 73% of comparative gene expression studies showing greater and more continuous immune activity in the more susceptible host (Eskew *et al*., 2021). For example, upon infection with chytridiomycosis, a fungal disease affecting amphibians, the susceptible wood frog (*Rana sylvatica*) demonstrates a more robust immune response compared to the resistant American bullfrog (*Rana catesbeiana*) (Eskew *et al*., 2018). Similarly, in a mouse model of Ebola virus, less susceptible individuals demonstrate a brief period of immune reactivity without a prolonged inflammatory response, while more susceptible individuals show signs of continued immune dysregulation (Price *et al*., 2020). Multiple hypotheses exist for the mechanism behind this phenomenon, but the underlying causes remain unclear.

Arguably the most devastating disease demonstrating this pattern of increased immune reactivity in susceptible species is white-nose syndrome (WNS), a disease impacting hibernating North American bats (Cheng *et al*., 2021; Hoyt *et al*., 2021). Infection is caused by the fungal pathogen, *Pseudogymnoascus destructans*, which grows on the exposed epidermal tissue of susceptible bat species during hibernation (Meteyer *et al*., 2009; Lorch *et al*., 2011). *Pseudogymnoascus destructans* destroys healthy bat tissue, forming cupping lesions as hyphae spread through the host epithelia (Meteyer *et al*., 2009). Tissue damage triggers a series of physiological disruptions that impact natural hibernation patterns (Cryan *et al*., 2010; Willis *et al*., 2011; Warnecke *et al*., 2013; Verant *et al*., 2014). Notably, *P. destructans* infection causes bats to arouse from torpor more frequently, which prematurely expends energy and water reserves and typically results in death (Cryan *et al*., 2010; Reeder *et al*., 2012).

The disturbance of hibernation physiology is thought to be caused, at least in part, by systemic inflammation and immune dysregulation. The immune response of bats is suppressed during torpor, allowing *P. destructans* to grow virtually unhampered across exposed epidermis (Meteyer *et al*., 2012; Field *et al*., 2018; Whiting-Fawcett *et al*., 2021). However, susceptible bat species appear to launch an aggressive immune response during periodic arousals (Field *et al*., 2018). While host immune responses are a primary defence against invading pathogens, they can become detrimental (Schulert and Grom, 2015) as seen in little brown bats (*Myotis lucifugus*). These observed responses are thought to exemplify pathological immune dysregulation (Hoyt *et al*., 2021; Whiting-Fawcett *et al*., 2021; Whiting-Fawcett *et al*., 2024), which presents as systemic inflammation that ultimately triggers the increased torpor arousal frequency indicative of WNS (Meteyer *et al*., 2012; Hoyt *et al*., 2021; Whiting-Fawcett *et al*., 2021). Supporting this idea, after spring emergence bats exhibit symptoms similar to immune reconstitution inflammatory syndrome in humans, where the sudden resurgence in immune activity leads to rampant systemic inflammation and often death (Meteyer *et al*., 2012).

Transcriptomic studies have provided specific insight into the reaction of bats during periodic arousals and suggest that infected bats mount an innate immune response typical of mammalian fungal dermatitis. Cell surface receptors respond to molecular identifiers from the pathogen itself and from tissue destruction caused by infection, stimulating secretion of cytokines and other immune signalling molecules (Moore *et al*., 2013; Rapin *et al*., 2014; Field *et al*., 2015; Lilley *et al*., 2017; Field *et al*., 2018; Davy *et al*., 2020; Whiting-Fawcett *et al*., 2021). These molecules then induce other aspects of the immune response involved in inflammation, such as mast cell degranulation and macrophage migration (Moore *et al*., 2013; Rapin *et al*., 2014; Field *et al*., 2015; Field *et al*., 2018). Such reactions also trigger a reduced adaptive immune response, as evidenced by a partial Th1 response, increases in Th17 and anti-*P. destructans* antibodies in infected wing tissue and neutrophil migration (Rapin *et al*., 2014; Lilley *et al*., 2017; Field *et al*., 2018; Whiting-Fawcett *et al*., 2021). Th1 and Th17 are both helper T lymphocytes that are activated by innate immune signalling after pathogen recognition, with Th1 being most notably related to responses to intracellular pathogens and Th17 to extracellular pathogens (Ouyang *et al*., 2008; Luckheeram *et al*., 2012). Unfortunately, this hampered adaptive response does not appear to effectively reduce *P. destructans* growth (Moore *et al*., 2011; Lilley *et al*., 2017; Lilley *et al*., 2019; Whiting-Fawcett *et al*., 2021).

The local inflammatory response associated with *P. destructans* growth appears to cause a systemic response in infected bats. Indeed, systemic inflammation and immune activity have been found in the lungs (Rapin *et al*., 2014) and epithelium (Davy *et al*., 2020) of infected individuals despite a lack of active pathogen growth in these areas. This systemic inflammatory response is hypothesized to cause the hibernation disruption indicative of WNS (Cheng *et al*., 2021; Hoyt *et al*., 2021; Whiting-Fawcett *et al*., 2021), although the mechanism responsible is not clearly understood. Multiple, non-mutually exclusive hypotheses have been proposed linking wing infection to mortality, including hypercapnia (excessive CO_2_ blood levels), dehydration and electrolyte imbalance (Cryan *et al*., 2010; Willis *et al*., 2011; Warnecke *et al*., 2013; Verant *et al*., 2014). However, the connection between systemic inflammation and these physiological abnormalities has not been directly demonstrated, especially given that most of the immune response appears to occur during arousals from torpor (Field *et al*., 2018).

Despite the plethora of information previously garnered about WNS pathophysiology, understanding of how host molecular response to disease directly causes the observed disruption of hibernation physiology remains incomplete. Considering how aspects of WNS pathophysiology relate to named conditions in humans and domestic animals may help elucidate how the immune response impacts physiological parameters. Here, we perform a differential gene expression analysis comparing wing tissue transcription patterns between little brown bats before and after exposure to *P. destructans* to identify immune genes that exhibit altered regulation. We combine our findings with those of previous transcriptomic work to generate hypotheses regarding mechanisms of WNS pathophysiology.

## Materials and Methods

### Bat wing tissue samples

**Figure 1:**
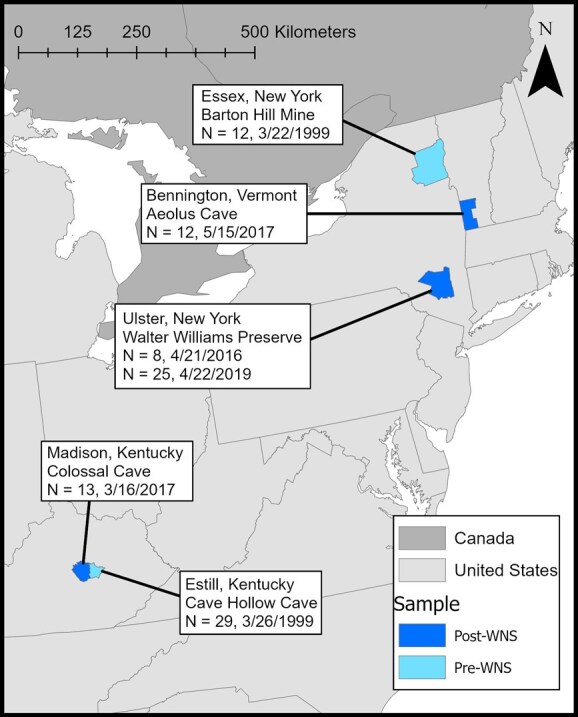
A map of the Eastern United States showing the counties from which samples were collected.

To represent gene expression patterns typical of little brown bats prior to *P. destructans* exposure, we obtained wing tissue samples of little brown bats collected prior to WNS emergence (1999) from two hibernation sites (hereafter, pre-WNS): Barton Hill Mine in New York and Cave Hollow Cave in Kentucky (Fig. 1, Supplementary Table 1). Samples were collected from torpid bats during the hibernation season, immediately preserved in 5 M NaCl with 20% dimethyl sulfoxide (DMSO), and subsequently stored at −80°C. To represent gene expression patterns of exposed little brown bat populations, we collected 58 samples from WNS-infected sites supporting persisting bat colonies in 2016, 2017 and 2019, and which had confirmed WNS presence for at least 2 years (hereafter, post-WNS). Exact pairing of pre- and post-WNS samples from the same sites was not possible due to logistical challenges; we therefore chose three post-WNS sites in close geographic proximity (<330 km) to each pre-WNS site: Walter Williams Preserve Mine (New York), Aeolus Cave (Vermont), and Colossal Cave (Kentucky; Fig. 1, Supplementary Table 1). Previous studies of little brown bats suggest little population structure at this scale, rendering it unlikely that differences in expression among hibernacula would be driven by genetic differences among populations (Miller-Butterworth *et al*., 2014; Johnson *et al*., 2015; Vonhof *et al*., 2015; Kwait *et al*., 2024). We collected post-WNS samples from torpid, hibernating bats by gently removing individuals from the hibernaculum substrate, extending a wing, and using a sterile biopsy punch to collect a 3-mm sample from the wing. We immediately placed wing tissue samples into 1.5-ml microcentrifuge tubes filled with RNAlater (Ambion Inc., Austin, TX), transported them to Rutgers University and stored them at −80°C. All samples were collected from random locations across the plagiopatagium and not guided by ultraviolet transillumination as has been done in other transcriptomic studies (Lilley *et al*., 2019; Davy *et al*., 2020). Random sampling occludes information about local versus systemic responses but allows for a larger sample size and the ability to use previously collected samples. All post-WNS samples were collected in close coordination with relevant state wildlife agencies under the appropriate permits and approvals (Rutgers IACUC Protocol #: 999900205). In all, we collected 41 samples from individuals prior to the emergence of WNS and 58 samples 2–5 years after WNS detection. We then generated 3′ poly-A enriched Tag-seq libraries from all samples.

### Transcriptomic library generation

We halved each wing biopsy punch with a scalpel, sterilizing between samples via ethanol burn (Kwait *et al*., 2024). We also sterilized the cutting board with 10% bleach between each sample. From one half of each sample, we extracted RNA using the RNeasy Micro kit (Qiagen, Hilden, Germany) following the manufacturer’s instructions, with DNase digestion performed in tube. To prepare these libraries for high-throughput sequencing, we used the Quant-seq 3’ mRNA-seq FWD kit (Lexogen, Wien, Austria) following the manufacturer’s protocol. This procedure starts with reverse transcription of RNA into cDNA followed by rRNA removal. We then amplified, added adapters and added sample-specific indexes to libraries using a polymerase chain reaction (PCR) that targets the poly-A tails of mRNA transcripts. Following amplification, we performed a magnetic bead purification and size selection using the protocol and reagents supplied with the Quant-seq kit. We then quantified libraries using the Qubit dsDNA high-sensitivity library quantification kit (Invitrogen, MA, USA). After preparation, we pooled samples in equimolar ratios into two batches and performed single-end sequencing on two lanes of an Illumina NovaSeq SP v1.5 with a read length of 100 bp (Kwait *et al*., 2024).

### Bioinformatics

Raw reads passing the quality filter were demultiplexed using BARCODESPLITTER and downloaded to the Amarel computer cluster at Rutgers University (Leach and Parsons, 2019). For all samples, we removed residual rRNA using the *bbtools* package (Bushnell, 2014) with the SILVA reference rRNA libraries SSU and LSU, which contained curated rRNA sequences from multiple taxa including bacteria and humans (Quast *et al*., 2012). We used *TRIMMOMATIC* (Bolger *et al*., 2014) to remove beginning and end bases with quality scores <3, sections with group quality scores <15 (sliding window 4:15), poly-A tails, adapters and trimmed reads <36 bp. We then removed reads that aligned to sequences of human, mouse, bacteria, fungi and viruses in GenBank using *FastQ Screen* (Wingett and Andrews, 2018). Filtered reads were aligned to the reference *M. lucifugus* genome (myoluc2.0) using *STAR aligner* (Dobin *et al*., 2013). The *STAR aligner* specifically accounts for spliced alignments, allowing for more accurate mapping of transcriptome-derived data (Dobin *et al*., 2013). Because pre- and post-WNS samples were preserved in different media, we compared raw read counts, percentage of uncalled bases, percentage of reads mapped, average mapped lengths and mapping mismatch percentages between sample groups to ensure they were of comparable quality (Kwait *et al*., 2024).

We generated count tables from read alignments using *HTSeq* (Anders *et al*., 2015) including the GFF file from the Myo_Luc2.0 reference genome and concatenated the read tables into one file. Using the R package *dplyr* (Ihaka and Gentleman, 1996; Wickham *et al*., 2015), we removed transcripts with counts <99 (the number of samples in the study) across all samples as well as those that did not have at least 10 counts in at least one sample. We then normalized the filtered libraries using the median of ratios method in *DESeq2* (Love *et al*., 2014).

### Differential expression analysis

We used the *DESeq2* package in R to perform a differential expression analysis comparing samples collected before and after WNS emergence (Love *et al*., 2014), first constructing a global model including WNS status (pre- or post-WNS), sequencing batch, sex and region of origin as covariates. For region of origin, we divided source hibernacula into two groups based on proximity, those sourced from the northeastern United States (New York and Vermont) and those from the southeastern United States (Kentucky). We then performed model selection to determine which covariates were informative using the likelihood ratio test as implemented in *DESeq2* (Love *et al*., 2014). This test compares nested models to determine how many individual gene model fits are significantly impacted by exclusion of a covariate. We considered a covariate informative if removal resulted in significant changes (FDR < 0.05) in the fit of more than 5% of gene models. Hereafter, FDR or false discovery rate refers to a Benjamini–Hochberg adjusted *P*-value. To this end, we first compared the global model to a reduced model that included WNS status, sequencing batch and region of origin as covariates but excluded the variable sex. If the first variable removed was found to be uninformative, we then compared a further reduced model excluding another variable to the previously reduced model. We repeated this procedure until our model only contained variables found to be informative. This methodology is similar to past uses of the likelihood ratio test in differential expression analyses (McCarthy *et al*., 2012; Love *et al*., 2014). Stepwise selection procedures such as the likelihood ratio test can be biased based on the order of variables removed (Whittingham *et al*., 2006). To account for the arbitrary choice of which variable to remove first, we performed multiple iterations of the modelling process while changing the order of variable selection.

Once we determined which covariates were informative, we performed a differential expression analysis comparing samples collected pre- and post-WNS, considering genes significantly differentially expressed if they exhibited a >4-fold change in expression and had an FDR < 0.001. We then performed a gene ontogeny (GO) term analysis on the significantly differentially expressed genes using the web server programme *g:profiler* with the g:GOSt function running default parameters and an FDR significance threshold >0.05 (Raudvere *et al*., 2019). The GO term analysis searched for functional enrichment among groups of input genes by searching for gene ontogeny terms that are overrepresented in comparison to a reference group of genes (Raudvere *et al*., 2019). We performed two separate GO term analyses for genes that were significantly upregulated and downregulated in WNS-exposed samples to determine if any were enriched relative to terms associated with all annotations of the reference little brown bat genome.

### Principal component regression

We also used a principal components analysis (PCA) to compare pre- and post-WNS samples, allowing us to identify genes that explained the most variation in our data and associate them with pre- versus post-WNS samples. We first performed a PCA on the normalized transcript counts using the *prcomp* function in R. We then extracted, standardized and centred the values of the first two principal components for each sample. We determined the best model distribution by creating a group of generalized linear models using each principal component as the response variable and WNS status as the categorical predictor assuming different distributions, including normal, gamma and inverse gamma. We specifically chose to test gamma and inverse gamma distributions to account for the left-skew of the data. In distributions requiring positive values only, we added an integer to each PC value to make the lowest value positive (+1 for PC1 and +2 for PC2). Once we determined the best fitting distribution, we prepared a global model to quantify the effect of disease presence on PC values using the principal components as the response variables and WNS status, sex, sequencing batch and region of origin (northeast or southeast) as covariates. From the global model, we created a set of models representing every combination of these covariates. Finally, we ranked models by AICc and averaged all models with a DAICc <2 using *MuMIn* (Burnham and Anderson, 2002; Barton and Barton, 2015). In this case, AICc refers to the Second-order Akaike Information Criterion as calculated by the package *MuMIn*. For quality control, we assessed model fit, dispersion, outlier influence and residual quantile similarity using the packages *car* and *DHARMa* in R (Fox *et al*., 2007; Hartig and Hartig, 2017). We then extracted the degree of correlation of each gene to each PC that was found to significantly differ between pre- and post-WNS samples. Genes with correlation values >0.5 were considered important to the PC. For all highly correlated genes, we then assessed function based on the annotation in GeneCards (Rebhan *et al*., 1998). All data manipulation and plots were created in R using the packages *dplyr*, *ggplot2*, *DHARMa* and *ggbiplot* (Ihaka and Gentleman, 1996; Vu, 2011; Wickham, 2014; Wickham *et al*., 2016; Hartig and Hartig, 2017).

#### Quantification of *P. destructans* reads

To verify infection status in post-WNS individuals, we quantified the number of reads mapping to the *P. destructans* genome. We filtered reads as described above but retained reads mapping to fungi from GenBank and instead filtered out reads mapping to the *M. lucifugus* or *Myotis myotis* genomes during the *fastqscreen* step. Then we mapped the remaining reads to the *P. destructans* genome in GenBank (GCF_001641265.1) using STAR aligner and filtered reads as above. Additionally, we removed genes with matches to the pre-WNS samples, making the assumption they were non-specific among local fungi and therefore, false positives. We then calculated the relative abundance of *P. destructans* reads per sample as the total number of reads aligning to the *P. destructans* genome divided by the total number of reads aligning to either the *P. destructans* or the *M. lucifugus* genome in each sample.

We then regressed the *P. destructans* reads against the principal components of post-WNS samples to determine if the relative quantity of pathogen RNA impacted the expression of *M. lucifugus* genes related to the principal components. We performed this analysis in R as described above for the principal component regression, using the relative abundance of *P. destructans* reads, sex, batch and region of origin as covariates.

## Results

After final filtering, we retained 25 853 817 reads with 261 150 ± 8673 (mean ± SEM) reads per sample. Metrics suggested sequences were of adequate quality with 0.18% ± 0.003 uncalled bases, a unique mapping rate of 76.57% ± 0.91, average mapped length of 89.37 bp ± 0.70 and alignment mismatch rate of 0.89% ± 0.01. The pre- and post-WNS sample quality comparison suggested the samples were comparable with similar mapping quality, read count and number of uncalled bases (Supplementary Table 2). After all filtering steps, 8014 expressed genes remained in the transcriptomic data.

**Figure 2:**
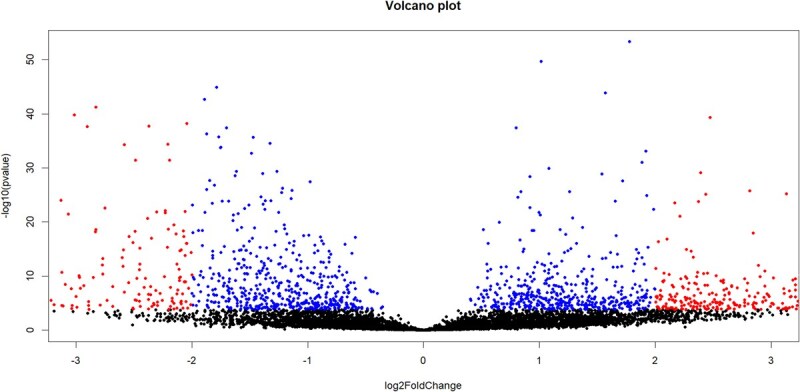
Volcano plot showing the results of the differential expression analysis. The *x*-axis shows the log 2-fold change of each gene between pre- and post-WNS samples and the *y*-axis is the -log10(FDR) of each relationship.

For model comparison as part of the differential expression analysis, we considered any covariate that could be excluded without impacting >401 gene models (5% of 8014) to be uninformative. Model selection suggested that both WNS status and sequencing batch affected differential gene expression patterns, while sex and region of origin did not and therefore were excluded from the final model (Supplementary Table 3). These same covariates were considered informative regardless of the order in which they were tested. In the final model, 375 genes had a >4-fold change in expression and an FDR < 0.001 between pre- and post-WNS samples (Fig. 2). Among the significantly differentially expressed genes, 235 were upregulated and 140 were downregulated in surviving exposed populations compared to unexposed populations (Supplementary Table 4). The upregulated genes had 22 significantly over-represented GO-terms, including several associated with cell death and cell motility. In addition, the terms ‘cellular response to cytokine stimulus’ and ‘response to stress’ were enriched among upregulated genes (Table 1A, full results in Supplementary Table 5). Downregulated genes in exposed individuals had 10 significantly enriched GO-terms, which were composed of general cellular structures and processes (i.e. intracellular anatomical structure, nucleus and intracellular membrane-bound organelle) (Table 1B).

**Figure 3:**
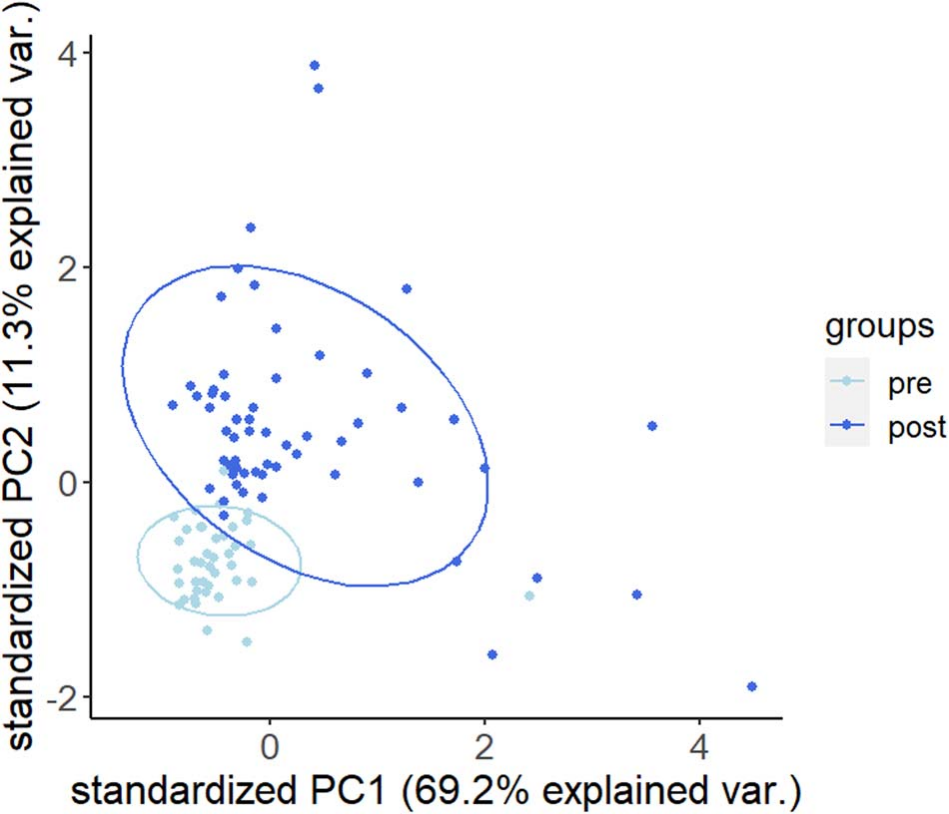
PCA biplot showing the first two standardized principal components grouped by WNS status.

**Table 1:** Significant GO terms found among differentially expressed genes using g:profiler

**GO term**	**FDR**	**Gene #**
**A**		
Cell migration	0.0002	27
Enzyme inhibitor activity	0.0002	14
Membrane protein complex	0.0002	24
Molecular function inhibitor activity	0.0016	14
Cell motility	0.0023	27
Organic substance transport	0.0049	30
Apoptotic process	0.0050	27
Peptidase regulator activity	0.0097	10
Cell death	0.0110	27
Programmed cell death	0.0110	27
Cellular response to cytokine stimulus	0.0111	16
Peptidase inhibitor activity	0.0132	9
Regulation of cell migration	0.0162	18
Enzyme regulator activity	0.0177	22
Nitrogen compound transport	0.0189	25
Regulation of programmed cell death	0.0246	23
Response to stress	0.0275	41
Regulation of cell motility	0.0381	18
Response to cytokine	0.0394	16
Positive regulation of cellular process	0.0411	56
Regulation of apoptotic process	0.0432	22
Cytoplasm	0.0487	86
**B**		
Intracellular anatomical structure	0.0007	92
MutSalpha complex	0.0084	2
Nucleus	0.0103	64
Single thymine insertion binding	0.0166	2
Intracellular membrane-bounded organelle	0.0174	78
Elg1 RFC-like complex	0.0250	2
Membrane-bounded organelle	0.0293	79
ATP hydrolysis activity	0.0487	8
Guanine/thymine mispair binding	0.0498	2
Single guanine insertion binding	0.0498	2

**Figure 4:**
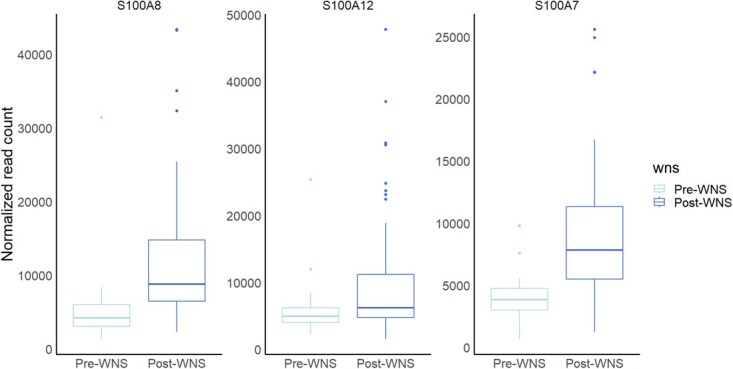
Boxplots comparing the normalized read counts of the genes most correlated with the first two principal components between samples collected pre- and post-WNS. The midline represents the median, the hinges show the first and third quartiles and the whiskers extend from the first and third quartiles to 1.5 × the interquartile range.

In the PCA, the first two principal components explained 80.5% of variation in gene expression, with PC1 alone explaining 69.2% (Fig. 3). For both PC1 and PC2, we used a generalized linear model assuming a gamma distribution with a log link function because that produced the lowest AIC_c_ of the distributions we evaluated. Top models for both PC1 and PC2 all included WNS status (Supplementary Table 6, Supplementary Table 7), and both PC1 (0.89 [0.61, 1.18]) and PC2 (0.69 [0.55, 0.83]) were significantly higher in post-WNS samples (Table 2). Sequencing batch appeared in the top model sets for both variables but was only significant for PC1. Model quality control analyses suggested good model fit and little overdispersion, with one of three statistics suggesting slight overdispersion in the PC2 model being the lone exception (Supplementary Fig. 1, Supplementary Fig. 2). The analytical results do not change appreciably when employing a normal distribution, but model fit assuming a gamma distribution is better (Supplementary Table 8, Supplementary Fig. 3). Two genes, S100A8 (0.70) and S100A12 (0.66), were highly correlated with PC1, while another S100 gene, S100A7, was highly correlated with PC2 (0.80). All three genes showed increased expression in post-WNS samples (Fig. 4).

**Table 2:** Model averaged results for model sets using PC1 and PC2 as response variables

Model set	Variable	Estimate	*P*-value
PC1	Intercept	−0.99 [−1.26, −0.72]	<0.0001
PC1	WNS Status (Post-WNS)	0.89 [0.61, 1.18]	<0.0001
PC1	Batch (seq2)	0.66 [0.37, 0.95]	<0.0001
PC1	Sex (M)	0.08 [−0.18, 0.35]	0.536
PC2	Intercept	0.24 [0.13, 0.36]	<0.0001
PC2	WNS Status (Post-WNS)	0.69 [0.55, 0.83]	<0.0001
PC2	Batch (seq2)	−0.02 [−0.11, 0.08]	0.725

Model set shows whether the statistics are for models that used PC1 or PC2 as the response variable. Estimate is the model average-derived coefficient with 95% confidence intervals for each variable. The variables listed for each model set are those that were included in models <2 ∆AICc of the top model.

The number of reads aligning to the *P. destructans* genome after filtering averaged 421 ± 68 (mean ± SEM) and comprised 0.15% ± 0.03 of aligned reads on average. The *P. destructans* reads detected among samples ranged from 0 to 2249 with the high end of that range accounting for 0.98% of aligned reads in that sample. We did not find a statistically significant relationship between either principal component with the proportion of reads aligning to the *P. destructans* genome, nor any of the other covariates with the exception of sequencing batch (Supplementary Table 9).

## Discussion

Our work has revealed significant differential gene expression patterns between little brown bats naïve to and persisting with WNS, adding detail to previous work describing the host transcriptional response to *P. destructans* infection. These observed patterns suggest upregulation of immune-related genes and gene families, including antigen processing and presentation, the NF-kb pathway, cytokine signalling and inflammatory response. These same pathways were identified as central to WNS pathophysiology in past work (Field *et al*., 2015; Davy *et al*., 2017; Lilley *et al*., 2017; Field *et al*., 2018; Lilley *et al*., 2019; Davy *et al*., 2020), suggesting our methodology is sufficient to detect transcriptional differences related to *P. destructans* infection.

Notably, the S100A7 gene was upregulated in persisting little brown bat populations compared to unexposed populations, supporting findings of past work demonstrating S100A7 activity in bat tissue with active fungal infection (Lilley *et al*., 2019). S100A7 impacts the differentiation of skin keratinocytes and responds to damage within the protective barrier of the epidermis (Gläser *et al*., 2009; Rangaraj *et al*., 2017). Upregulation of S100A7 directly impacts wound healing and is a transcriptional marker differentiating chronic, unhealed wounds from those that heal properly (Andresen *et al*., 2011; Rangaraj *et al*., 2017). The S100A7 protein also has antimicrobial activity and is typically overexpressed in proliferative skin diseases, potentially in support of its relationship to wound healing (Meyer *et al*., 2008; Gläser *et al*., 2009). In bats infected with *P. destructans*, upregulation of S100A7 may promote the healing process or relate to an inability to heal lesions associated with fungal growth. Alternatively, or in concert, S100A7 expression may reduce the likelihood of secondary infection after fungal disruption of the skin barrier given its antibacterial properties.

Multiple over-represented GO-terms were associated with cellular functions that are difficult to link directly to WNS yet have come up in past studies. For example, four terms related to cell motility or migration and three related to organelles were over-represented (Table 1A). Although it is possible that these processes relate to disease, especially given they have been identified in other studies of WNS-induced transcriptional changes (Field *et al*., 2015; Davy *et al*., 2017; Field *et al*., 2018; Lilley *et al*., 2019), their broad functional activity makes inference uncertain. Past authors have suggested the upregulation of general cellular functions such as these may be a sign of increased metabolic demand of the immune response (Field *et al*., 2015; Davy *et al*., 2017; Lilley *et al*., 2019).

### The role of cell death in WNS

Among genes that were upregulated in post-WNS samples, we found five over-represented GO-terms associated with the process of apoptosis, which has been related to infectious disease in humans and mice (Labbe and Saleh, 2008; Riera Romo, 2021). For viral or intracellular pathogens, apoptosis facilitates pathogen load reductions via cell destruction (Labbe and Saleh, 2008; Riera Romo, 2021). However, apoptosis of specific cells can be induced by and benefit the invading pathogen. For example, the bacterium *Bacillus anthracis* causes cellular death of macrophages to avoid being phagocytized (Hanna *et al*., 1993). Cellular damage resulting from pathogen infiltration can also lead to programmed death of impacted cells (Labbe and Saleh, 2008). Further, programmed cell death occurs in some immune cells (i.e. neutrophils) after they perform their function, and elevated levels of immune cell death occur in cases of immune dysregulation during uncontrolled infection (Riera Romo, 2021).

Potentially associated with a rise in cell death, we found increased expression of two damage-associated molecular patterns (DAMPs: S100A8 and S100A12) in bats from populations persisting with WNS. Released from cells after death (Wang *et al*., 2018), DAMPs are immune signalling molecules hypothesized to be part of the response of little brown bats to WNS (Whiting-Fawcett *et al*., 2021). The proteins S100A8 and S100A12 are S100 proteins similar to S100A7; however, instead of being sequestered in and released by epithelial cells, S100A8 and S100A12 are often released by white blood cells (Rosen *et al*., 2022). These proteins have previously been associated with the little brown bat response to infection (Field *et al*., 2018; Lilley *et al*., 2019), likely serving to stimulate the immune system by acting as ligands for toll-like receptors (Vogl *et al*., 2007; Foell *et al*., 2013; Jackson *et al*., 2017; Wang *et al*., 2018). In other infections, this signalling initiates local inflammatory responses by stimulating the secretion of cytokines and other pro-inflammatory factors (Nacken *et al*., 2003; Ryckman *et al*., 2003; Yang *et al*., 2007; Wang *et al*., 2018; Guo *et al*., 2021). Further, S100A8 occurs in high quantities within neutrophils and is released upon death of the cells after performing their function (Hessian *et al*., 1993; Ryckman *et al*., 2003). In turn, signalling by S100A8 stimulates the release of cytokines that cause the chemotaxis of more neutrophils (Ryckman *et al*., 2003; Wang *et al*., 2018). We see further support for this pathway in the over-represented GO-terms ‘cytokine stimulus’ and ‘cellular response to cytokine stimulus’ among genes that were upregulated in exposed samples, suggesting an increase in cytokine expression. When the neutrophil response is continually ineffective in limiting pathogen growth, this cell death–DAMP–cytokine pathway could lead to a feedback loop of amplifying immune activity. Increased neutrophil activity has been observed in bats with WNS (Courtin *et al*., 2010; Rapin *et al*., 2014; Whiting-Fawcett *et al*., 2021), suggesting a neutrophil death–DAMP–cytokine cascade could be an important component of disease progression.

The role of cell death in WNS may be further complicated by recent evidence demonstrating that *P. destructans* conidia can survive and proliferate within bat keratinocytes by blocking apoptosis (Isidoro-Ayza and Klein, 2024). This intracellular life cycle is predicted to help the pathogen escape anti-fungal immune responses during torpor arousal. In that same study, there was no evidence of an increase in DAMP expression, suggesting infiltration occurred with little damage to the invaded cell or that DAMP expression was directly suppressed by *P. destructans* (Isidoro-Ayza and Klein, 2024). These findings may indicate that a large portion of the DAMP- and apoptosis-related expression patterns we observed do not originate from keratinocytes but rather from different cell types, such as other epithelial cells or immune cells.

### Relationships to other pathologies

Inflammation in infected little brown bats and its pathological effects are well supported (Meteyer *et al*., 2012; Field *et al*., 2018; Lilley *et al*., 2019; Davy *et al*., 2020). Observed inflammation is likely caused by expression of pro-inflammatory cytokines and genes related to that pathway, such as toll-like receptors and NF-kb (Meteyer *et al*., 2012; Field *et al*., 2018; Lilley *et al*., 2019; Davy *et al*., 2020). Our results also include upregulation of the processes involved in cytokine release in exposed little brown bats. The immune reconstitution inflammatory syndrome-like response infected bats suffer after spring emergence is also likely the result of a release of pro-inflammatory cytokines (Meteyer *et al*., 2012; Whiting-Fawcett *et al*., 2021). Most major systemic inflammatory syndromes are the result of the uncontrolled release of cytokines (Jaffer *et al*., 2010). Although cytokine release during infection is a natural part of the immune response, it can become pathological and cause a cytokine storm (Tisoncik *et al*., 2012). For example, the SARS CoV-2 virus responsible for COVID-19 is often not fatal and can be successfully cleared by the human immune system with time (Ragab *et al*., 2020). However, extreme overproduction of cytokines in some individuals can cause systemic inflammation and multiple organ dysfunction syndrome (Ragab *et al*., 2020; Hu *et al*., 2021; Zanza *et al*., 2022).

Although the presence of cytokines does not imply a cytokine storm, prolonged infections often result in such dysregulation (Jaffer *et al*., 2010; Tisoncik *et al*., 2012; Schulert and Grom, 2015). It is worth noting that a cytokine storm may be avoided during the hibernation season because some aspects of the immune response are suppressed during torpor (Field *et al*., 2018; Whiting-Fawcett *et al*., 2021), potentially decreasing the duration of cytokine activity. Also, the majority of the post-WNS samples we included in this study had some concentration of *P. destructans* RNA, so it is difficult to ascertain if the expression patterns observed here result from a local or a systemic response more akin to a cytokine storm. However, we found no relationship between the amount of *P. destructans* RNA in a wing biopsy and expression levels of the S100 genes, suggesting the local *P. destructans* load alone does not predict immune activity. In addition, given *P. destructans* infection typically lasts the duration of the hibernation season (Langwig *et al*., 2015) and that some bats undergo immune reconstitution inflammatory syndrome after emergence (Meteyer *et al*., 2012), it is likely that some individuals experience systemic overexpression of cytokines as part of a continuously ineffective immune response during the hibernation season.

In other taxa, a continuous cytokine storm can eventually deteriorate into sepsis amidst uncontrolled disease. Sepsis is defined as immune dysregulation and multiple organ failure caused by underlying prolonged infection (Singer *et al*., 2016; Schlapbach *et al*., 2024). Interestingly, the same neutrophil death–DAMP–cytokine feedback loop discussed above is a significant component of sepsis (Cheng *et al*., 2015; Dubois *et al*., 2019; Cheng *et al*., 2020; Sehgal *et al*., 2022). Elevated levels of S100A8 and S100A12 are considered a potential biomarker of sepsis in human patients, and their levels of expression have been associated with poor disease outcomes (Payen *et al*., 2008; Dubois *et al*., 2019; Sehgal *et al*., 2022). Further, increased cell death, especially of immune cells such as lymphocytes, has been directly linked to sepsis in humans and other mammals (Dubois *et al*., 2019; Cheng *et al*., 2020). Lymphocyte activity and subsequent apoptosis increases through the early progression of sepsis, and its magnitude is predictive of patient outcomes in clinical settings (Le Tulzo *et al*., 2002; Roth *et al*., 2003; Efron *et al*., 2004; Wesche *et al*., 2005; Lang and Matute-Bello, 2009). Our findings, many of which are supported by previous studies, may indicate sepsis in bats with severe cases of WNS due to immune dysregulation and prolonged infection.

In addition, many WNS symptoms are shared with sepsis. Even on the surface the similarities are quite clear: immune dysregulation is a hypothesized morbidity in WNS (Lilley *et al*., 2017; Field *et al*., 2018; Lilley *et al*., 2019; Davy *et al*., 2020; Whiting-Fawcett *et al*., 2021; Whiting-Fawcett *et al*., 2024), and the infection is prolonged over the majority of the hibernation season (Langwig *et al*., 2015). Both WNS and sepsis also include fever, metabolic changes, hyperventilation, bone marrow granulocytosis and systemic inflammation (Courtin *et al*., 2010; Meteyer *et al*., 2012; Verant *et al*., 2014; Cheng *et al*., 2015; Edman-Wallér *et al*., 2016; Polat *et al*., 2017; Field *et al*., 2018; Cheng *et al*., 2021; Hoyt *et al*., 2021; Whiting-Fawcett *et al*., 2021; Guenther *et al*., 2024). Therefore, further investigation into the presence and role of sepsis in WNS is warranted.

If sepsis is part of WNS pathology, underlying strain on organs and eventual failure has potential to impact the physiological parameters associated with torpor arousal frequency in bats. Indeed, sepsis has been associated with several metabolic symptoms hypothesized to result in arousal, including dehydration (Thapa *et al*., 2013; Long and Koyfman, 2017), electrolyte imbalance (Ahmad *et al*., 2018) and in some cases hypercapnia if the lungs are affected (Tiruvoipati *et al*., 2022). However, many of these symptoms could also be the result of starvation and dehydration caused by the increased frequency of torpor arousal. Despite that possibility, the same increased transcriptomic immune responses in susceptible species in bats exist in other species that do not hibernate (Eskew *et al*., 2021). It is possible these uncontrolled infections, such as those in amphibians with chytridiomycosis and in bats with WNS, lead to cytokine storms due to continuous ineffective immune activity that can deteriorate into sepsis in late stages of disease. Less susceptible species would then avoid mortality by either successfully combating infection (resistance) or not inducing an immune response despite infection (tolerance), as has been suggested in less susceptible bats and amphibians (Eskew *et al*., 2018; Lilley *et al*., 2019; Davy *et al*., 2020; Cheng *et al*., 2024; Whiting-Fawcett *et al*., 2024). However, a sepsis diagnosis requires more validation, typically utilizing multiple biochemical and immunohistochemical analyses for certainty (Long and Koyfman, 2017; Stassi *et al*., 2020).

### Implications

Consideration of sepsis as part of the WNS pathology is prudent, given recent advances in the medical field that suggest sepsis may have meaningful consequences for disease management. Physicians have recognized that widespread inflammation associated with sepsis may be more adaptive than previously acknowledged, and organ failure associated with infection is more likely a direct cause of morbidity (Singer *et al*., 2016). This revelation may be germane to WNS research as much attention has been given to the systemic inflammatory response, but little has been given to the possibility of multiple organ failure, at least from the perspective of failure caused by immune dysregulation (but see Blehert *et al*., 2009; Courtin *et al*., 2010). It is possible we are currently missing organ failure as an important part of WNS pathophysiology.

Determining if sepsis is part of WNS progression could also reveal potential therapeutics. For example, mice treated with an S100A8 neutralizing agent were protected from systemic inflammation and fatal immune dysregulation in a model of cytokine storms associated with COVID-19 (Guo *et al*., 2021). Treatment with this same agent, paquinimod, has potential in reducing pathological immune dysregulation in bats with WNS. Alternatively or in addition, cytokine-directed therapies have shown promise in treatment of systemic inflammatory syndrome (Schulert and Grom, 2015) and may be applicable to bats. However, translating such treatment strategies to a relatively distantly related, free-living species such as bats can be challenging and therefore requires validation prior to implementation.

Our findings also suggest continued immune dysregulation in some little brown bats from surviving populations in the northeast. These populations have begun exhibiting signs of positive population growth following initial mass mortality caused by WNS (Hoyt *et al*., 2021; Maslo *et al*., 2015; Reichard *et al*., 2014). This change is thought to be, in part, due to physiological changes such as increased fall fat mass (Cheng *et al*., 2019) and reduction of the pathological increase in torpor arousal frequency despite continued pathogen infection (Lilley *et al*., 2016; Frank *et al*., 2019). These hypotheses are supported by genomic changes in little brown bats implying rapid evolution resulting in increased survival (Donaldson *et al*., 2017; Auteri and Knowles, 2020; Gignoux-Wolfsohn *et al*., 2021). However, our results suggest that immune dysregulation still occurs in a significant number of individuals. These findings mirror a previous study demonstrating a maladaptive immune response despite escape from large population declines in little brown bats (Lilley *et al*., 2019). The previous study also showed that the immune response to *P. destructans* was altered, though still likely maladaptive, in surviving populations compared to naïve populations of little brown bats (Lilley *et al*., 2019). Here, we found evidence of substantial intraspecific variation in immune responses within WNS-exposed bat populations. Post-WNS samples show high variability compared to pre-WNS samples in expression of the S100 genes, with many individuals clustering closer to pre-WNS expression levels and some showing large deviation (Fig. 4). Supporting such intraspecific variation, some of the genomic loci putatively under selection due to WNS appear to regulate the expression of innate immune genes, suggesting a genetic basis for intraspecific variation in immune response (Gignoux-Wolfsohn *et al*., 2021; Kwait *et al*., 2024). Alternatively, or in concert, the individual variation in gene expression observed here may result from variable disease severity, with more immune dysregulation in individuals with more intense infection. Lastly, necropsies of WNS-infected bats showed that inflammation was most common in individuals afflicted with a secondary bacterial infection around fungal penetrations into tissue (Courtin *et al*., 2010), implying variation in disease severity can result from co-infection. Altogether, this suggests that intraspecific variation in disease severity in little brown bats likely depends both on genetics and environmental variables including bacterial coinfection.

The observed changes in transcription patterns in persisting populations of little brown bats (Lilley *et al*., 2019), some of which are based on genetic differences (Kwait *et al*., 2024), suggest that some of the patterns uncovered here may be different in naïve bat populations. Some parts of the little brown bat range, including the regions sampled in this study, are considered to be in the endemic phase of WNS, implying lower disease severity and mortality in these populations (Frank *et al*., 2019; Cheng *et al*., 2021) in part due to rapid evolution (Gignoux-Wolfsohn *et al*., 2021). Therefore, some of the transcription changes we observed may be adaptive rather than examples of immune dysregulation. For example, increased expression of S100A7 could relate to successful wound healing in little brown bat populations that have adapted and/or acclimated to WNS but would not occur in naïve populations that have yet to adapt.

### Limitations

Existing study limitations can also inform interpretation of our results. First, our pre- and post-WNS samples were not paired from the same hibernacula and were collected from different regions of North America (NE and SE), making population structure a potentially confounding variable. Although pre/post-WNS samples were often geographically close, we cannot fully control for site-level factors (i.e. microclimate) that could have impacted gene expression. However, region of origin was not a statistically significant factor in any of our analyses. In addition, previous studies of population structure in little brown bats suggest panmixia across their range (Dixon, 2011; Johnson *et al*., 2015; Vonhof *et al*., 2015; Wilder *et al*., 2015), and DNA-based analyses of our specific samples showed no population structure according to geography (Kwait *et al*., 2024). In addition, we did not use UV illumination to target areas of *P. destructans-*induced wing damage. Therefore, we cannot confirm whether wing biopsies represented areas of active pathogen growth, complicating our ability to differentiate a local and systemic response. However, the random sampling design we employed allowed us to acquire a large sample size consisting entirely of wild-caught bats. Many previous studies of WNS transcriptomics occurred in a laboratory setting, and wild-caught individuals may respond differently to disease. Also, almost all post-WNS samples in this study had a detectable level of *P. destructans* RNA and previous work suggests near-100% prevalence of *P. destructans* on individuals from infected sites (Hoyt *et al*., 2020), strongly implying infection in these samples.

Finally, this analysis is confounded by sample age and storage condition (NaCL with DMSO for pre-WNS samples vs RNALater for post-WNS samples). It is possible that either of these factors could drive differences in observed gene expression patterns that make the true WNS-related signal difficult to disentangle. To confront this issue, we included analyses of sequencing quality between our sample groups and found that they were comparable in terms of quality (Supplementary Table 2), although this does not capture potential differences in RNA composition. These factors have the potential to impact our results; however, given the relevance of our findings to infectious disease and the concordance of most patterns we detected with previous studies, we believe it is most probable that the differential expression signals we report are attributable to WNS.

## Conclusion

We completed a differential expression analysis comparing the wing tissue transcription patterns between bats from populations persisting with WNS and those collected from hibernacula prior to disease emergence. Our results offer complementary validation of previous findings suggesting that bats infected with *P. destructans* undergo innate immune activation followed by an inflammatory response. We also found evidence of expression changes related to skin lesions/dermatitis and the process of wound healing including the activity of the gene S100A7. Additionally, we see evidence of a feedback loop between immune cell death, the release of DAMPs (S100A8 and S100A12) and cytokine stimulation, leading to increased immune cell activity and inflammation. This loop supports the role of pathological immune dysregulation in WNS and may lead to a cytokine storm, and potentially sepsis, in late-stage disease progression. Sepsis as a pathology of WNS would suggest underlying multiple organ dysfunction, a relationship that may prove crucial in connecting the immune response to the altered physiology of WNS-affected bats. To our knowledge, this is the first suggestion of potential cytokine storms and sepsis in bats with WNS. Considering sepsis as part of WNS pathophysiology might have a meaningful impact on understanding the symptoms of WNS and potential treatment options, although further validation is required for any type of diagnosis.

## Supplementary Material

Web_Material_coaf040

## Data Availability

Raw sequences are publicly available from the NCBI Short Read Archive (BIOProjectID PRJNA1115790). The sequences used in this work include those from Kentucky, New York and Vermont in the single read library of that BIOProject.
