# Supplementary material for: Transcriptomic evidence of cytokine storm and sepsis in little brown bats exposed to white-nose syndrome
Source: Conserv Physiol. 2025 Jul 1;13(1):coaf040. doi: 10.1093/conphys/coaf040 (PMC12212055; doi:10.1093/conphys/coaf040)
Supplement: Web_Material_coaf040 [file web_material_coaf040.zip › Supplemental.pdf]

State	Pre or Post WNS	WNS Infection Year	Sample #	Hibernaculum	Collection Date
New York	Post	~2006-2010	25	Walter Williams Preserve	04/22/2019
New York	Post	~2006-2010	8	Walter Williams Preserve	04/22/2016
Vermont	Post	~2007-2009	12	Aeolus Cave	05/15/2017
Kentucky	Post	~2012-2015	13	Colossal Cave	03/16/2017
New York	Pre	~2007-2009	12	Barton Hill Mine	03/22/1999
Kentucky	Pre	~2013	29	Cave Hollow Cave	03/26/1999

Supplementary Table 1. Hibernacula from which samples were collected and the dates of collection.

Period	Sample Size	Raw read count	N%	Mapped%	Mapped Length	Mismatch%
Pre-WNS	41	$4.5X10^6 \pm 4.1X10^5$	0.19 ± 0.005	80.37 ± 9.00	89.89 ± 7.64	0.88 ± 0.10
Post-WNS	58	$5.5X10^6 \pm 4.6X10^5$	0.17 ± 0.005	73.76 ± 8.22	88.99 ± 6.63	0.90 ± 0.10

Supplementary Table 2. Quality metrics of the raw sequencing reads. Raw read count refers to the number of sequencing reads before filtering. N% refers to the number of uncalled bases after sequencing. Mapped % refers to the number of uniquely aligned reads. Mapped Length is the average length in base pairs of mapped reads during alignment. Mismatch% is the alignment mismatch rate. All reported statistics are mean ± SEM.

Variable	Gene models impacted
Sex	0-1
Region of origin	244-245
Sequencing batch	685-776
WNS status	1996-2856

Supplementary Table 3. Model comparison results from DESeq2 likelihood ratio test. The Gene models impacted column indicates the range of the number of gene models whose fit significantly (FDR < 0.05) changed after exclusion of the associated variable when removing the variables in different orders. We considered a variable uninformative if its exclusion impacted fewer than 5% of genes. Variables denoted in gray are those considered uninformative by that criterion.

Supplementary Table 4. (Attached as Excel file). Full results table of differential expression analysis as output by DESeq2.

Supplementary Table 5. (Attached as Excel file). Full results table of GO-term analysis as output by g:profiler.

(Intrc)	batch	reg	sex	wns	df	loglike	AICc	delta	weight
-9.53E-01	+			+	4	-62.722	133.9	0	0.407
-1.04E+00	+		+	+	5	-61.783	134.2	0.34	0.343
-9.35E-01	+	+		+	5	-62.694	136	2.16	0.138
-1.03E+00	+	+	+	+	6	-61.776	136.5	2.6	0.111
-8.54E-01			+	+	4	-73.161	154.7	20.88	0
-9.85E-01		+	+	+	5	-72.066	154.8	20.91	0
-6.31E-01				+	3	-76.481	159.2	25.34	0
-7.61E-01		+		+	4	-75.397	159.2	25.35	0
-4.11E-01	+	+	+		5	-77.581	165.8	31.94	0
-2.41E-01	+	+			4	-79.342	167.1	33.24	0
-5.22E-01	+		+		4	-81.206	170.8	36.97	0
-3.33E-01	+				3	-83.392	173	39.17	0
-3.14E-01			+		3	-89.712	185.7	51.81	0
-2.90E-01		+	+		4	-89.62	187.7	53.8	0
3.09E-12					2	-93.642	191.4	57.54	0
1.31E-02		+			3	-93.624	193.5	59.63	0

Supplementary Table 6. AIC table for models including PC1 as a response variable. Reg refers to region of origin (Northeast or Southeast), batch refers to sequencing lane, and WNS refers to the variable WNS status (pre- or post-WNS). A + in the variable columns indicates which variables were used in each model. DF refers to the degrees of freedom.

(Intrc)	batch	reg	sex	wns	df	logLik	AICc	delta	weight
0.2344				+	3	-106.66	219.6	0	0.389
0.2579	+			+	4	-106.431	221.3	1.71	0.165
0.2481			+	+	4	-106.612	221.6	2.08	0.138
0.2497		+		+	4	-106.634	221.7	2.12	0.135
0.2626	+		+	+	5	-106.422	223.5	3.92	0.055
0.253	+	+		+	5	-106.427	223.5	3.93	0.055
0.2602		+	+	+	5	-106.593	223.8	4.26	0.046
0.2577	+	+	+	+	6	-106.418	225.7	6.18	0.018
0.8249		+			3	-127.461	261.2	41.6	0
0.757		+	+		4	-126.833	262.1	42.52	0
0.8052	+	+			4	-127.045	262.5	42.94	0
0.7523	+	+	+		5	-126.616	263.9	44.3	0
0.6931					2	-133.161	270.4	50.87	0
0.7299	+				3	-132.741	271.7	52.16	0
0.6422			+		3	-132.875	272	52.43	0
0.6717	+		+		4	-132.272	273	53.4	0

Supplementary Table 7. AIC table for models including PC2 as the response variable. Reg refers to region of origin (Northeast or Southeast), and WNS refers to the variable WNS status (pre- or post-WNS). A + in the variable columns indicates which variables were used in each model. DF refers to the degrees of freedom.

Model Set	Variable	Estimate	p-value
PC1	Intercept	-0.78 [-1.13, -0.44]	<0.0001
PC1	WNS Status (Post-WNS)	0.81 [0.43, 1.18]	<0.0001
PC1	Batch (seq2)	0.69 [0.33, 1.06]	0.000191
PC1	Sex (M)	0.03 [-0.19, 0.25]	0.756
PC1	Region of Origin (SE)	0.04 [-0.21, 0.28]	0.773
PC2	Intercept	-0.70 [-0.99, -0.41]	<0.0001
PC2	WNS Status (Post-WNS)	1.25 [0.92, 1.58]	< 0.0001
PC2	Batch (seq2)	-0.07 [-0.34, 0.20]	0.610
PC2	Region of Origin (SE)	-0.01 [-0.18, 0.15]	0.867

Supplementary Table 8. Model averaged results for model sets using PC1 and PC2 as response variables assuming a normal distribution. Model set shows whether the statistics are for models that used PC1 or PC2 as the response variable. Estimate is the model average derived coefficient with 95% confidence intervals for each variable. The variables listed for each model set are those that were included in models < 2 ΔAICc of the top model.

Model Set	Variable	Estimate	p-value
PC1	Intercept	-0.11 [-0.55, 0.33]	0.63
PC1	Batch (seq2)	0.91 [0.26, 1.55]	0.005
PC1	Region of Origin (SE)	0.09 [-0.45, 0.63]	0.73
PC1	Sex (M)	0.06 [-0.30, 0.42]	0.74
PC1	Relative PD Load	10.42 [-63.74, 84.58]	0.78
PC2	Intercept	0.64 [0.28, 0.99]	0.0003
PC2	Batch (seq2)	-0.32 [-0.98, 0.35]	0.34
PC2	Region of Origin (SE)	0.05 [-0.36, 0.46]	0.82

Supplementary Table 9. Model averaged results for model sets using PC1 and PC2 as response variables. Model set shows whether the statistics are for models that used PC1 or PC2 as the response variable. Estimate is the model average derived coefficient with 95% confidence intervals for each variable. Relative PD Load refers to the proportion of aligned reads mapped to the *P. destructans* genome. The variables listed for each model set are those that were included in models < 2 ΔAICc of the top model.

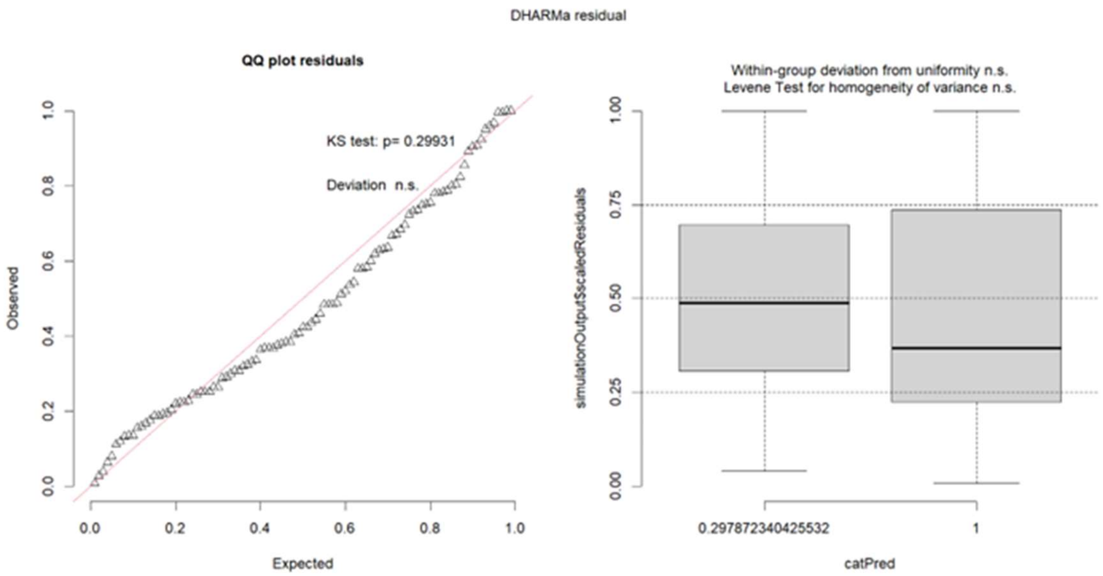

Supplementary Figure 1. DHARMa package qq and residuals plots for model using PC1 as a response variable and WNS status as a predictor.

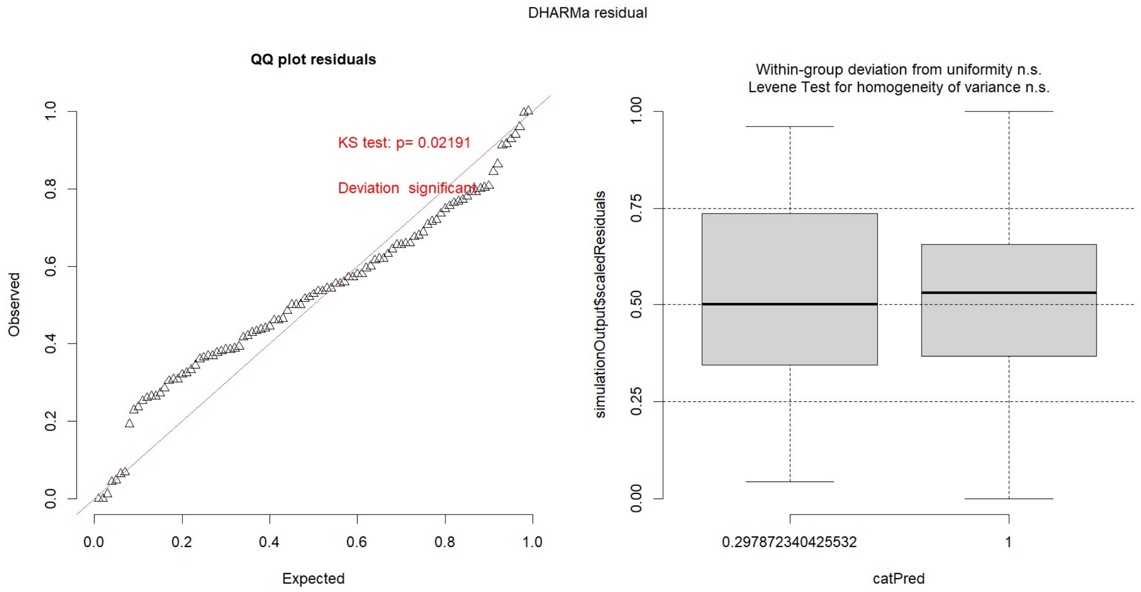

Supplementary Figure 2. DHARMa package qq and residuals plots for model using PC2 as a response variable and WNS status as a predictor.

A

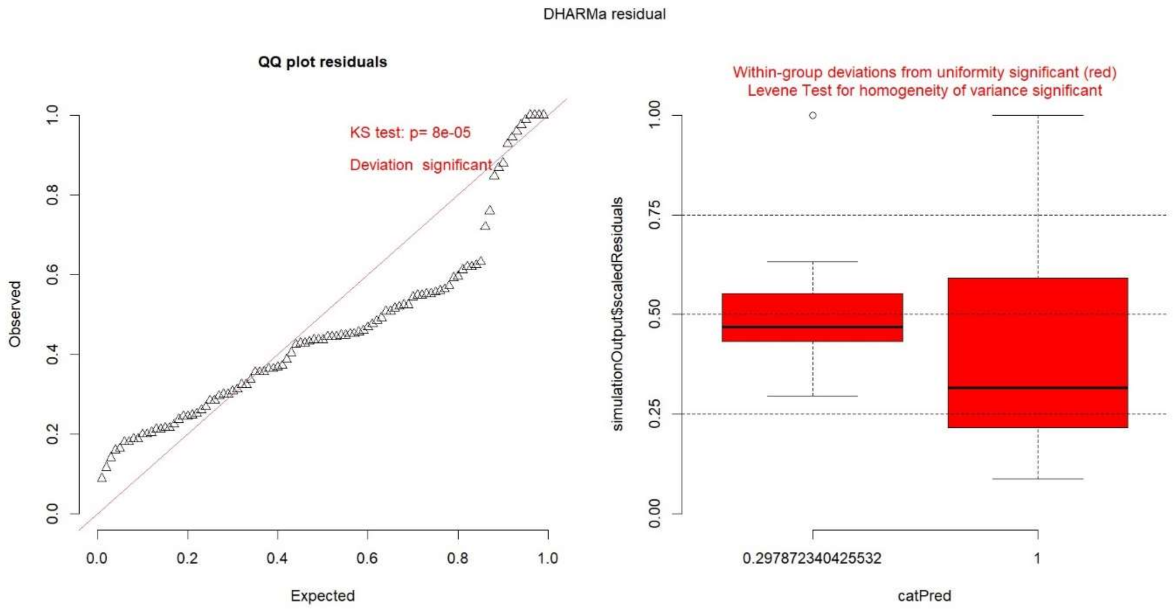

B

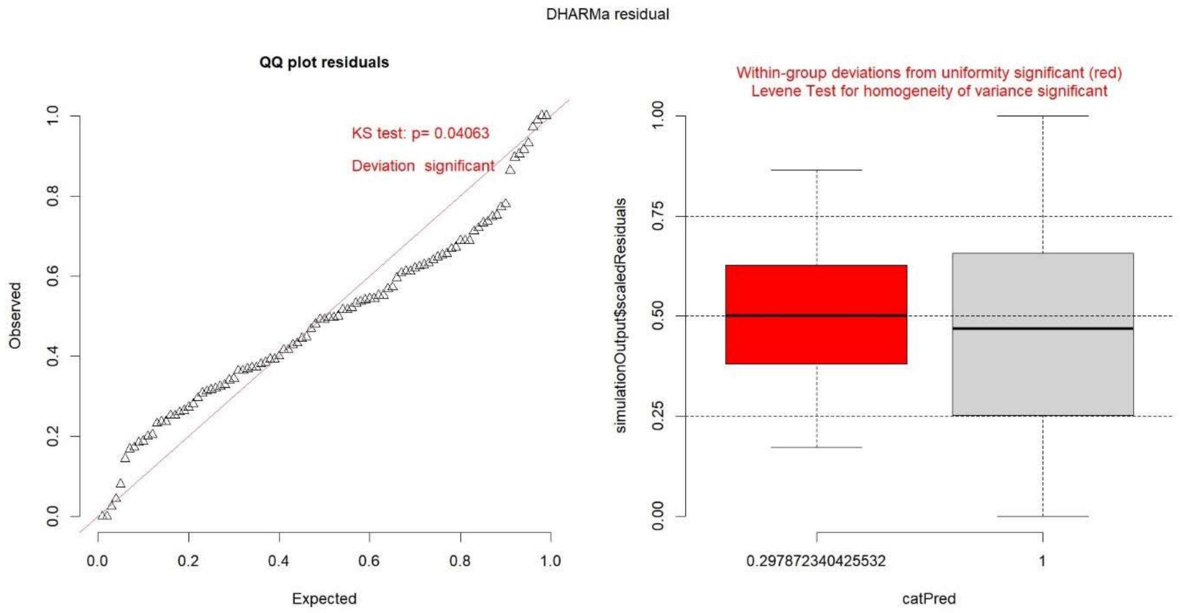

Supplementary Figure 3. DHARMa package qq and residuals plots for model employing a normal distribution using A) PC1 and B) PC2 as a response variable and WNS status as a predictor.
